# Coronary–bronchial artery fistula arising from the sinoatrial nodal artery in a patient with bronchiectasis: a multimodality imaging case report

**DOI:** 10.1093/ehjcr/ytag501

**Published:** 2026-07-14

**Authors:** Maria Nieves Montoro-Lopez, Gisela Feltes, Ángel Domínguez Álvarez, Iván J Núñez-Gil

**Affiliations:** Cardiology Department, Hospital Universitario de Torrejón, Ribera Salud Group, Calle Mateo Inurria, Torrejón de Ardoz, Madrid 28850, Spain; Cardiology Department, Hospital Universitario de Torrejón, Ribera Salud Group, Calle Mateo Inurria, Torrejón de Ardoz, Madrid 28850, Spain; Faculty of Medicine, Health and Sports, Department of Medicine, Universidad Europea de Madrid, Calle Tajo s/n, Villaviciosa de Odón, Madrid 28670, Spain; Radiology Department, Hospital Universitario de Torrejón, Ribera Salud Group, Calle Mateo Inurria, Torrejón de Ardoz, Madrid 28850, Spain; Cardiology Department, Hospital Universitario de Torrejón, Ribera Salud Group, Calle Mateo Inurria, Torrejón de Ardoz, Madrid 28850, Spain; Faculty of Medicine, Health and Sports, Department of Medicine, Universidad Europea de Madrid, Calle Tajo s/n, Villaviciosa de Odón, Madrid 28670, Spain

**Keywords:** Case report, Coronary–bronchial artery fistula, Sinoatrial nodal artery, Bronchiectasis, Coronary computed tomography angiography, Multimodality cardiovascular imaging, Coronary artery anomalies

## Abstract

**Background:**

Coronary–bronchial artery fistulas are rare vascular anomalies between the coronary and bronchial circulations. They are most often detected incidentally and may be congenital or acquired, particularly in the setting of chronic pulmonary disease. Origin from the sinoatrial nodal artery is exceptional. If haemodynamically significant, these communications may lead to a coronary steal phenomenon and represent a potential cause of myocardial ischaemia. Recognition is also crucial prior to bronchial artery embolization to avoid inadvertent coronary complications. Multimodality imaging plays a key role in their diagnosis and characterization.

**Case summary:**

A 58-year-old man with psoriasis presented with atypical chest pain. Exercise stress echocardiography was electrically equivocal, prompting invasive coronary angiography. No significant coronary artery disease was identified. However, contrast injection into the proximal right coronary artery demonstrated a prominent atrial branch with opacification of the right pulmonary parenchyma. Coronary computed tomography angiography revealed a retroaortic sinoatrial nodal artery originating from the proximal right coronary artery. Its left branch communicated with a hypertrophied bronchial artery, which followed a tortuous trajectory towards the right middle lobe. Chest computed tomography demonstrated cylindrical and varicose bronchiectasis in the right middle and lower lobes. No haemoptysis occurred.

**Discussion:**

This case illustrates a rare coronary–bronchial artery fistula arising from the sinoatrial nodal artery in association with bronchiectasis. Multimodality imaging enabled accurate anatomical characterization and suggested possible haemodynamic relevance. Such fistulas may represent a potential cause of myocardial ischaemia and should be recognized both in the evaluation of chest pain and prior to interventional procedures.

Learning points

- Coronary-to-bronchial artery fistulas should be considered in patients with chronic bronchiectasis and unexplained myocardial ischaemia.
- Multimodality imaging is key to establishing the diagnosis, assessing clinical relevance, and guiding treatment planning.

## Introduction

Coronary–bronchial artery fistulas are rare vascular connections between the coronary and bronchial circulations, most often detected incidentally during coronary imaging.^1,2^ They may be congenital or acquired, particularly in the setting of chronic pulmonary disease, where inflammatory and angiogenic mechanisms can promote systemic collateral recruitment.^3^ Although usually asymptomatic, these fistulas may occasionally be associated with a coronary steal phenomenon and represent a potential cause of myocardial ischaemia.^1^ Involvement of the sinoatrial nodal artery is exceptionally uncommon, and angiographic evidence suggesting a functional component has been only rarely described.

We report a case of a coronary–bronchial fistula arising from a retroaortic sinoatrial nodal artery in a patient with bronchiectasis, highlighting the role of multimodality imaging in anatomical characterization and its potential clinical implications.

## Summary figure

**Figure:**
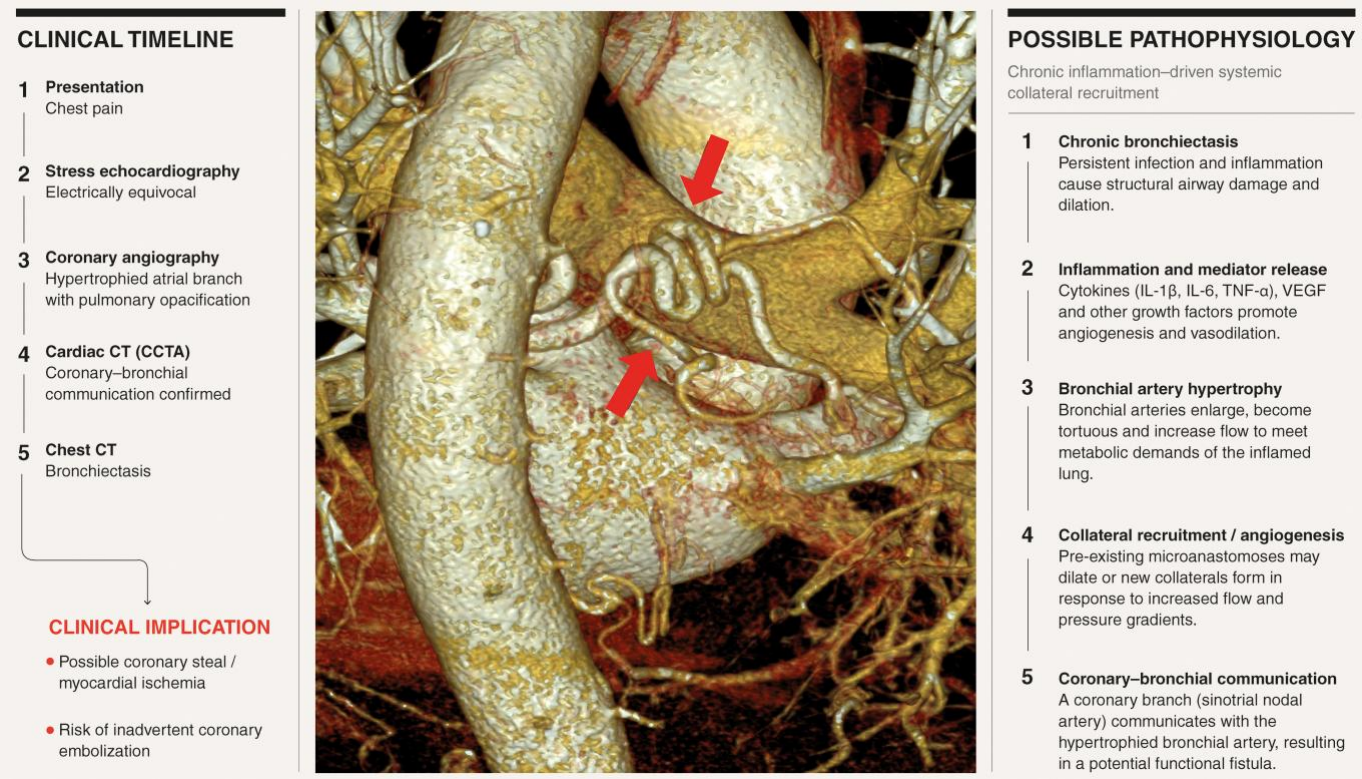
Acquired sinoatrial nodal artery-to-bronchial artery fistula: case timeline and pathophysiological overview. Right panel: clinical timeline summarizing the diagnostic work-up, from presentation to multimodality imaging confirmation. Central panel: volume-rendered coronary computed tomography angiography demonstrating the point of communication between a hypertrophied, tortuous bronchial artery (superior) and the sinoatrial nodal artery arising from the proximal right coronary artery (inferior, retroaortic course). Left panel: schematic representation of the most plausible pathophysiological mechanism, whereby chronic bronchiectasis promotes inflammation, angiogenesis, bronchial artery hypertrophy, and systemic collateral recruitment, potentially rendering coronary–bronchial fistulas.

## Case presentation

A 58-year-old Romanian male, a former smoker, with dyslipidaemia and psoriasis treated with methotrexate and folic acid, presented with localized left-sided chest pain with atypical features for angina. Blood pressure and physical examination were normal (*Summary figure*, left panel).

Electrocardiography showed right bundle branch block and left anterior fascicular block. Transthoracic echocardiography demonstrated normal biventricular size and function. Exercise stress echocardiography was clinically reproducible (>85% predicted heart rate), electrically equivocal due to repolarization changes in right precordial leads, and associated with recurrence of symptoms.

**Figure 1:**
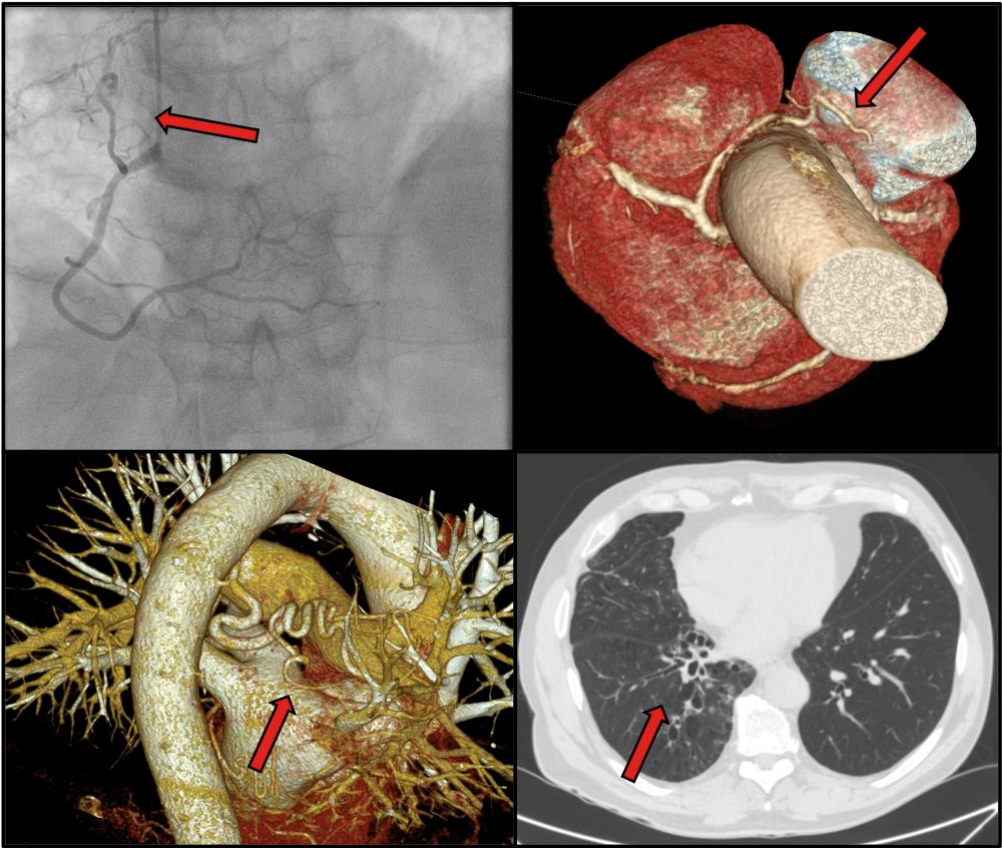
Multimodality imaging of a coronary–bronchial artery fistula. Upper left: invasive coronary angiography of the right coronary artery. The arrow indicates a hypertrophied atrial branch arising from the proximal segment and coursing posteriorly. Opacification of the right pulmonary parenchyma after contrast injection suggests communication with pulmonary or bronchial collateral vessels. Upper right: volume-rendered coronary computed tomography angiography. An atrial branch with a retroaortic course is visualized, showing distal bifurcation. The arrow indicates the branch converging with the bronchial artery along the superior aspect of the left atrium. Lower left: volume-rendered coronary computed tomography angiography demonstrating a hypertrophied bronchial artery originating from the anterior surface of the descending thoracic aorta and following a tortuous course towards the right bronchial tree. The arrow indicates the convergence between the atrial branch and the hypertrophied bronchial artery. Lower right: chest computed tomography showing cylindrical and varicose bronchiectasis in the right middle and lower lobes, with mucous plugging and radiological findings suggestive of superinfection.

Coronary angiography showed no obstructive coronary artery disease (*Figure 1*, upper right). However, a prominent atrial branch arising from the proximal right coronary artery demonstrated contrast passage towards the right pulmonary parenchyma, suggestive of an extracardiac vascular communication.

Coronary computed tomography (CT) angiography, performed using a retrospectively electrocardiogram-gated acquisition protocol with intravenous contrast administration, confirmed the presence of a retroaortic sinoatrial nodal artery originating from the proximal right coronary artery (*Figure 1*, upper left). After coursing posterior to the ascending aorta and reaching the interatrial septum, the artery bifurcated. The left branch travelled along the superior surface of the left atrium and converged with a hypertrophied bronchial artery, measuring 4.7 mm in proximal diameter, arising from the descending thoracic aorta (*Summary figure*, central panel). The bronchial artery followed a tortuous course towards the right middle lobe (*Figure 1*, lower left).

Chest CT demonstrated cylindrical and varicose bronchiectasis in the right middle and lower lobes with mucous plugging and imaging signs suggestive of inflammation and superinfection (*Figure 1*, lower right).

Bronchoscopy with bronchoalveolar lavage was negative for malignancy and did not identify bacterial, fungal, or mycobacterial pathogens. However, given the radiological findings suggestive of bronchiectasis with possible superinfection and the clinical context, the patient was treated with empirical antibiotic therapy and clinical follow-up, with subsequent improvement in symptoms.

Although exertional symptoms and equivocal repolarization changes during stress testing raised the possibility of myocardial ischaemia, the findings were not conclusive. In the absence of haemoptysis, signs of volume overload, or definitive evidence of ischaemia, and given the clinical improvement after treatment of bronchiectasis, a conservative strategy with clinical and radiological surveillance was adopted.

## Discussion

Coronary artery fistulas represent abnormal communications between coronary arteries and cardiac chambers or vascular structures. Coronary–bronchial artery fistulas are an uncommon subtype, reported in ∼0.5%–0.6% of patients undergoing coronary imaging.^1,2^ Most originate from the left circumflex artery, whereas an origin from the right coronary artery is less frequent. Involvement of the sinoatrial nodal artery is particularly rare.^4^ Although most coronary artery fistulas are considered congenital,^5^ acquired forms have also been described in association with chronic pulmonary disease and systemic inflammatory conditions.^3^

A limited number of cases involving the sinoatrial nodal artery have been reported, including isolated case reports and small series. In the largest CT-based series, sinoatrial nodal branch involvement was identified in 9 of 15 patients with coronary–bronchial arterial communications.^6^ Individual case reports have also described this rare anatomical origin in association with bronchiectasis and other pulmonary conditions.^3,4^ However, angiographic evidence suggesting contrast passage to the pulmonary parenchyma—indicative of a potentially functional component—has been reported only rarely.^3,7^ Therefore, the present case adds to the limited literature by demonstrating a coronary–bronchial artery communication with angiographic contrast passage suggestive of a functional component.

It is important to distinguish between a purely anatomical coronary–bronchial fistula and a functionally relevant shunt. The latter may lead to a coronary steal phenomenon, whereby blood flow is diverted from the coronary circulation towards a lower-resistance bronchial vascular bed. This mechanism has been described as a potential cause of myocardial ischaemia, particularly during increased myocardial demand.^1,8^ In the literature, chest pain and dyspnoea are among the most commonly reported symptoms, suggesting that these communications may represent a potential and possibly underappreciated cause of myocardial ischaemia, particularly in patients with chronic inflammatory lung disease, such as bronchiectasis.

In the present case, the presence of a coronary–bronchial fistula was supported by clear anatomical delineation on both invasive angiography and coronary computed tomography angiography. Angiographic opacification of the pulmonary parenchyma following contrast injection suggests passage beyond the coronary circulation and indirect communication with the bronchial vascular bed. However, this finding remains indirect and does not provide definitive evidence of haemodynamic significance. No temporal analysis of contrast transit, quantitative flow assessment, or perfusion imaging (such as stress cardiac magnetic resonance or nuclear imaging) was available to confirm myocardial ischaemia. Therefore, a coronary steal phenomenon related to the coronary–bronchial communication could not be entirely excluded. Nevertheless, the absence of high-risk features on stress testing, together with clinical improvement following treatment of bronchiectasis and the lack of haemoptysis, supported a conservative management strategy with clinical and imaging follow-up.

Two main mechanisms have been proposed for the development of coronary–bronchial fistulas (*Summary figure*, left panel): persistence or enlargement of congenital anastomoses and acquired angiogenic recruitment in the setting of chronic pulmonary inflammation.^1,6,9^ Chronic inflammatory lung diseases, including bronchiectasis, tuberculosis, cystic fibrosis, and other suppurative airway disorders, are associated with bronchial arterial hypertrophy and systemic collateral recruitment.^10^ Recurrent airway infection and inflammation may promote local vasodilatation, endothelial activation, and angiogenesis, with vascular endothelial growth factor and other pro-angiogenic pathways contributing to bronchial vascular remodelling.^11,12^ In this setting, pre-existing microvascular anastomoses between systemic, bronchial, and coronary circulations may enlarge, or new collateral pathways may develop, making an otherwise silent anatomical communication angiographically apparent. In our case, the coexistence of right-sided bronchiectasis, mucous plugging, a hypertrophied and tortuous bronchial artery, and angiographic pulmonary parenchymal opacification supports this acquired inflammatory-remodelling hypothesis. However, this remains inferential, and a congenital communication secondarily accentuated by chronic pulmonary inflammation cannot be excluded.

Reported indications for treatment of coronary–bronchial artery fistulas include significant or recurrent haemoptysis, documented myocardial ischaemia attributable to a coronary steal phenomenon, or evidence of haemodynamically relevant shunting.^13^ In such cases, treatment options may include bronchial artery embolization or, less commonly, surgical or percutaneous closure.^8^

Recognition of these fistulas remains clinically important. Bronchial artery embolization represents the standard treatment for haemoptysis in patients with bronchiectasis; however, unrecognized coronary–bronchial fistulas may result in inadvertent coronary embolization and myocardial infarction.^8,13^ Thus, current interventional standards emphasize careful evaluation of potential non-target vessels prior to embolization procedures.^13^ In contrast, in asymptomatic or minimally symptomatic patients without evidence of ischaemia or haemodynamic compromise, a conservative approach is generally more appropriate.^5^ However, non-intervention is not entirely risk-free. Although the natural history of coronary–bronchial fistulas is not well defined, these findings may potentially progress or become haemodynamically relevant over time. In our patient, a follow-up plan, including clinical reassessment and consideration of repeat imaging in case of symptom recurrence, was adopted.

Multimodality imaging played a key role in the present case. Coronary computed tomography angiography allowed precise delineation of the origin of the sinoatrial nodal artery, its retroaortic course, bifurcation, and convergence with the hypertrophied bronchial artery, thereby providing comprehensive anatomical characterization of both coronary and extracardiac vascular structures and facilitating clinical decision-making.^2,4,14^

In conclusion, this case highlights that coronary–bronchial fistulas may be identified during the evaluation of chest pain and underscores the importance of multimodality imaging for accurate anatomical characterization. These findings may be associated with a coronary steal mechanism and represent a potential cause of myocardial ischaemia. Recognition is also essential prior to thoracic or interventional procedures to avoid inadvertent coronary complications.

## Data Availability

The data supporting the findings of this case report are included within the article. Additional anonymized data are available from the corresponding author upon reasonable request.

## Lead author biography

Dr Maria Nieves Montoro-Lopez is a consultant cardiologist in Madrid, Spain. She trained in cardiology at Hospital Universitario La Paz and is currently part of the Advanced Cardiac Imaging Unit at Hospital Universitario de Torrejón, where she focuses on multimodality cardiac imaging and clinical cardiology. Her clinical interests include cardiovascular prevention, structural heart disease, and the role of imaging in complex cardiovascular conditions. She also holds a Master’s degree in Health Economics from the London School of Economics, reflecting her interest in the intersection between clinical cardiology, healthcare systems, and value-based medicine. She combines hospital practice with telemedicine and collaborative clinical research.

## Notes

**Consent:** The authors confirm that written consent for submission and publication of this case report including images and associated text has been obtained from the patient in line with the COPE guidelines.
